# Role of Natural Antioxidants on Neuroprotection and Neuroinflammation

**DOI:** 10.3390/antiox10040608

**Published:** 2021-04-15

**Authors:** Domenico Nuzzo

**Affiliations:** Consiglio Nazionale delle Ricerche, Istituto per la Ricerca e l’Innovazione Biomedica (CNR-IRIB), 90146 Palermo, Italy; domenico.nuzzo@cnr.it

All cells continuously generate reactive oxygen species (ROS) through the respiratory chain during the energy metabolism process. When ROS are maintained at low or moderate concentrations, they can play important physiological roles and serve as important second messengers in cell signaling. However, too many ROS are extremely harmful to live systems, and long-term exposure can damage key cellular macromolecules such as DNA, proteins and lipids, leading to necrotic and apoptotic cell death [1]. Oxidative stress condition is generated by an imbalance between the formation of reactive oxygen species and the cellular antioxidant capacity that can be attributed to increased ROS generation and/or to the dysfunction of the natural antioxidant system. Biochemical modifications in macromolecular components can lead to various human pathological conditions and diseases. Among all systems and organs, the central nervous system (CNS) is particularly vulnerable to oxidative stress due to its high oxygen consumption, weak antioxidant system, and characteristic terminal differentiation of neurons. For this reason, oxidative stress is considered to underlie many neurodegenerative diseases. Numerous cellular processes are altered during oxidative stress, such as mitochondrial regulation and function, DNA repair, cell signaling, and other fundamental physiological processes [1]. Specifically, mitochondrial dynamics are recognized to be a common feature of a wide range of neurodegenerative diseases. This process is strongly influenced by ROS production, mtDNA genetics and epigenetics, which act at the interface with environmental exposures. Antioxidant dietary intake can have positive effects on mitochondrial dynamics and brain cells [2]. In fact, many dietary antioxidants are highly efficient in contrasting oxidative damage, and act as free radical scavengers. Huge amount of vegetables, fruits and berries are a rich source of natural antioxidants, such as anthocyanins, biophenols, vitamins, and other phytochemicals, which are actively involved in slowing down the ageing process [3]. Recently, several phytochemical complexes with antioxidant function have been identified as potential therapeutic agents to prevent/treat neurodegeneration disorders [4,5,6,7]. Specifically, the ingestion of foods with high antioxidant power, such as nuts, can prevent neuronal dysfunctions during obesity [8].

Oxidative stress has long been considered one of the most important pathological mechanisms involved in Parkinson’s disease (PD). In fact, the association between PD and oxidative stress is confirmed by post-mortem analyses of PD patients’ brains, where increased oxidative activity and cellular damage were observed in dopaminergic neurons. Extensive research is focused on developing effective antioxidant systems as a promising therapy for this devastating disease. Some vitamins such as vitamin D, a natural water-soluble antioxidant, play significant roles as free radical scavengers. In fact, results in cellular models positively supported this theory. Integration with vitamin C had a neuroprotective effect against levodopa’s neurotoxicity in vitro. In addition, its implementation with other antioxidants preserved and enhanced the activity of the antioxidant system of neurons in preclinical models of PD [9]. Furthermore, in a recent article, Dr. Espinoza and collaborators demonstrated that vitamin C treatment improved neuronal differentiation. In their work, the authors described how vitamin C recycling is an important mechanism in regulating neuronal morphology during neuronal differentiation and maturation [10]. Several studies have shown that other nutraceutical compounds, such as carotenoids, polyphenols and omega-3s, when consumed daily, reduce the risks of chronic neurodegenerative diseases. In fact, it is now clear that natural antioxidant and anti-inflammatory molecules can constantly help in counteracting symptoms in people with autism spectrum disorders (ASD) [11]. In particular, it is important to highlight that these molecules are extremely useful for body health, as they can simultaneously fight inflammation and oxidative stress and reduce damage in several organs, including the brain. Numerous molecules with neuroprotective effects can be present in a specific part of the food. Mangosteen pericarp water extract (MPW) showed antioxidative neuroprotection and anti-apoptotic action in mice model for dementia. When MPW was administrated in scopolamine-treated mice, spatial memory function was improved [12]. Verbascoside, isolated from the leaves of *Acanthus mollis* L., a herbaceous plant of the Acanthaceae family, showed potential therapeutic effects thanks to its ability to inhibit MAO-A and, therefore, decrease the degradation of monoaminergic neurotransmitters. This effect is enhanced by its ability to scavenge free radicals and, consequently, decrease the oxidative stress induced in neuronal cells [13]. The essential oil of *Thymus vulgaris* is characterized by a large amount of monoterpenes, and the two main phenolic monoterpenes, thymol and carvacrol, occur more frequently. This natural oil exhibits potential effects for improving the cholinergic nervous system and oxidative protection in the Zebrafish model of cognitive dysfunction [14]. Chiaino and collaborators presented a paper in which a mix of the extracts obtained from *Olea europea* L. leaves and *Hybiscus sabdariffa* L. calyces were analyzed. On the SH-SY5Y cellular model and rat brain slices, the authors demonstrated that these extracts exhibited neuroprotective effects. They had a synergistic effect caused by the combination of their different components. Regular intake of phytochemicals could strengthen the antioxidant system, thus increasing the survival of neuronal cells and improving mental activity [15]. In a recent paper, the effect of *Erythronium japonicum* aqueous extract on brain inflammation was evaluated. In this study, the authors analyze anti-inflammatory and antioxidant effects on activated BV2 microglia. Their results were very interesting because the bioactive compounds showed a decrease in the level of inflammation and oxidative stress markers [16]. Natural antioxidants also have a positive effect in reducing memory loss and cognitive dysfunction. In fact, an extract of *Juglans regia* L. was able to reduce oxidative stress in brain tissue and improve cognitive function through the reinforcement of the anti-inflammatory response at the blood–brain barrier level [17].

In conclusion, it is now evident that diet and natural and nutraceutical compounds have a direct and indirect effect on the brain. Decreases in oxidative stress and inflammation are essential requirements for brain health. Nevertheless, it is important to keep in mind that results obtained in experimental models can be less effective when translated in clinical trials. Thus, further research should be undertaken to better understand the secrets of food.
